# Familial study revealed the association of Vitamin D receptor gene haplotype with Hansen’s disease

**DOI:** 10.1186/1471-2334-12-S1-P16

**Published:** 2012-05-04

**Authors:** NV Sanjeev, NC Suryadevara, Suman Jain, Vijaya Lakshmi Valluri, MPJS Anandraj

**Affiliations:** 1 LEPRA India – Blue Peter Public Health & Research Centre, Hyderabad, AP, India; 2 Thalassemia and Sickle Cell Anaemia Society (TSCS), Hyderabad, AP, India

## Background

Single-nucleotide polymorphism within the gene encoding Vitamin D receptor (VDR) – a member of the nuclear receptor supergene family, is associated with several infectious diseases. The receptor belongs to the family of trans-acting transcriptional regulatory factors. Studies on VDR gene polymorphism reveals Fok I, Taq I, & Apa I restriction site variants to be significantly associated with many of the diseases compared to other SNPs within the gene. The study aims to determine the association of these polymorphisms with Hansen’s disease.

## Methods

The study group included six well defined multicase leprosy families with cases (n=32) and unaffected family members (n=44). Genotyping was done for the polymorphic positions present in exon 2(T/C), 9(T/C) and intron 8(C/A) regions of the VDR gene using Polymerase Chain Reaction followed by Restriction Fragment Length Polymorphism using enzymes Fok I, Taq I & Apa I respectively. Haplotype analysis was performed for the three positions using Chi square test in SNPSTAT software.

## Results

Out of all possible combinations on haplotype analysis, C-T-C (*p*=0.018) and T-T-C (*p*=0.028) was negatively associated with Hansen’s disease and no significant association was observed with individual gene variants. The wild alleles at position Taq I and Apa I were found to be in strong linkage disequilibrium.

## Conclusion

The data indicates that a relationship exists between VDR polymorphic haplotype and the development of disease and the haplotypes C-T-C and T-T-C may perhaps render protection against Hansen’s disease.

